# A Sensor-Based Upper Limb Treatment in Hemiplegic Patients: Results from a Randomized Pilot Study

**DOI:** 10.3390/s24082574

**Published:** 2024-04-17

**Authors:** Fabio Vanoglio, Laura Comini, Marta Gaiani, Gian Pietro Bonometti, Alberto Luisa, Palmira Bernocchi

**Affiliations:** 1 Neuromotor Rehabilitation Unit of Institute of Lumezzane, Istituti Clinici Scientifici Maugeri IRCCS, 25065 Lumezzane, Italy; fabio.vanoglio@icsmaugeri.it (F.V.); marta.gaiani@icsmaugeri.it (M.G.); giampiero.bonometti@icsmaugeri.it (G.P.B.); alberto.luisa@icsmaugeri.it (A.L.); 2 Scientific Direction of Institute of Lumezzane, Istituti Clinici Scientifici Maugeri IRCCS, 25065 Lumezzane, Italy; laura.comini@icsmaugeri.it; 3 Continuity of Care Service of Institute of Lumezzane, Istituti Clinici Scientifici Maugeri IRCCS, 25065 Lumezzane, Italy

**Keywords:** hand function, sub-acute stroke, sensor-based device, upper extremity, rehabilitation

## Abstract

In post-stroke patients, the disabling motor deficit mainly affects the upper limb. The focus of rehabilitation is improving upper limb function and reducing long-term disability. This study aims to evaluate the feasibility of using the Gloreha Aria (R-Lead), a sensor-based upper limb in-hospital rehabilitation, compared with conventional physiotherapist-led training in subacute hemiplegic patients. Twenty-one patients were recruited and randomised 1:1 to a sensor-based group (treatment group TG) or a conventional group (control group, CG). All patients performed 30 sessions of 30 min each of dedicated upper limb rehabilitation. The Fugl–Meyer Assessment for Upper Extremity (FMA-UE) was the primary evaluation., both as a motor score and as individual items. Secondary evaluations were Functional Independence Measure; global disability assessed with the Modified Barthel Index; Motor Evaluation Scale for UE in stroke; power grip; and arm, shoulder, and hand disability. All the enrolled patients, 10 in the TG and 11 in the CG, completed all hand rehabilitation sessions during their hospital stay without experiencing any adverse events. FMA-UE scores in upper limb motor function improved in both groups [delta change CG (11.8 ± 9.2) vs. TG (12.7 ± 8.6)]. The score at T1 for FMA joint pain (21.8 vs. 24 best score) suggests the use of the Gloreha Aria (R-Lead) as feasible in improving arm function abilities in post-stroke patients.

## 1. Introduction

Stroke is one of the leading causes of death and disability worldwide [1]. Impairments and limitations of cognitive, language, perceptual, sensory, and motor functions with reduced ability to perform activities of daily living induced by the disease affect stroke survivors [2,3]. Hemiplegia is one of the most common symptoms in stroke patients, causing movement problems and, therefore, restrictions in daily activities [4,5]. After a stroke, the patient’s recovery can be significant within the first three months and then more slowly in the following year. In the first days, a reduction in oedema and partial reperfusion of the ischaemic penumbra may be the explanation for these phenomena. Still, improving the neurological deficit in the following weeks suggests plasticity phenomena and brain cortical reorganisation [6,7,8]. Restoring arm and hand skills after a stroke remains challenging due to the amount of work required, the availability of therapists, the length of rehabilitation sessions, and associated costs, although stroke rehabilitation programmes have proven their effectiveness [9]. In particular, repetitive task training has been proven to be effective in some aspects of rehabilitation, improving walking distance, speed, and upper limb function [10,11,12].

In post-stroke patients, the disabling motor deficit mainly affects the upper limb. The focus of rehabilitation is improving upper limb function and reducing long-term disability [13,14,15].

Robotic systems can offer repetitive and reproducible forms of physical therapy that are quantifiable. Studies have shown that robot-assisted treatment is safe, well-tolerated, and has a positive impact on motor impairments [16]. The use of robotic devices, providing passive and/or active movements, including repetitive, intensive, and task-oriented exercises, appears to reduce the motor deficit of the arm and affected hand and improve hand function both at the wrist and fingers [17,18,19,20,21,22].

Other than robotic therapy, virtual reality (VR) is also becoming increasingly used in the upper limb and hand rehabilitation of chronic strokes [23]. VR is an innovative approach to engaging and motivating patients. It can apply relevant neuroplasticity concepts, i.e., task-oriented, repetitive, and intensive training. It offers a new possibility to provide feedback on a patient’s performance by directly interconnecting motor behaviour with actions in the virtual scenario. Moreover, the simulation of activities through games that mix virtual stimuli, objects, and environments with real ones (augmented reality) in combination with conventional therapy [24,25] has been developed for post-stroke rehabilitation and proved to be effective for upper limb recovery [26].

In this study, the investigators employed a novel passive sensor-based device, Gloreha Aria (R-Lead), designed for the motor recovery of impaired upper limbs, coupled with VR.

Gloreha Aria (R-Lead) offers programmes that assist patients with the movement of their arms, wrists, and fingers, providing an interactive experience that enhances the real world with perceptual information generated by computers through games. Therapists can customise therapy by focusing on a specific motor task.

The main purpose of this pilot study was to evaluate the device feasibility, considering also pre- and post-evaluations of the upper arm movements in patients affected by sub-acute stroke.

## 2. Materials and Methods

The Ethics Committee approved the study on 29 May 2018 (approval n. 2208CE). The protocol followed the principles stated in the Declaration of Helsinki. All patients gave their written informed consent. This pilot prospective and randomised controlled study was registered on the ClinicalTrials.gov website as NCT03738813 on 8 November 2018. Post-stroke patients admitted for inpatient rehabilitation at Neuromotor Rehabilitation of the Istituti Clinici Scientifici (ICS) Maugeri, Lumezzane (Brescia) were screened for enrolment between June 2018 and October 2021.

Inclusion criteria were the following: age >18 years; patients hospitalised after a first event of cerebral vascular accident occurred ≤30 days before, with a unilateral hemispherical lesion confirmed by Computed Tomography (CT) or Magnetic Resonance Imaging (MRI); and exhibiting upper limb paresis with an Ashworth spasticity index of less than 3 [27].

Patients were excluded if they had the following: inability to understand verbal instructions or motor commands; unilateral neglect; significant visual impairment; unstable medical conditions; significant orthopaedic limitations in the shoulder, elbow, wrist, or hand; peripheral nerve lesions in the upper arm; neuromuscular or neurodegenerative diseases; and an Ashworth spasticity index ≥ 3.

### 2.1. Study Design

This was a pilot study that assessed patient compliance and side-effects with device use and physiotherapist judgement on the device. The study also evaluated the device’s performance in rehabilitating upper arm movement in patients with subacute strokes.

### 2.2. Patient Data Acquisition

At baseline (T0)

Participant characteristics: age, gender, BMI, main lesion site, affected side, and modified subtype of stroke [28].

At baseline (T0) and the end of the study (T1)

- Fugl–Meyer Assessment Upper Extremity (FMA-UE) was used to measure sensorimotor function (score 0–126) [29]. The assessment tool includes subscales for motor function, sensory function, range of motion, and joint pain. Each item is evaluated on a 3-point ordinal scale ranging from 0 (not performed) to 2 (smooth and complete performance).
  - Motor function assessment evaluates reflex activity and range of motion in the shoulder, elbow, forearm, wrist, and hand. It involves testing flexion, extension, and rotation in specific positions. The subscale consists of 24 items, with a score range of 0 to 66.
  - Sensation. This has six items, and the score for this subscale ranges from 0 to 12.
  - Range of motion and joint pain. This has 12 items, which are scored for each range of motion and joint pain. The score for this subscale ranges from 0 to 48.
  - For the present study, we considered the range of joint pain (score 0–24) as a further indicator of feasibility in TG. A higher FMA-UE score indicates less upper limb impairment.
- The degree of independence and need for assistance in the basic activities of daily living (ADL) were measured using the Functional Independence Measure (FIM), which includes both motor and cognitive subscales. FIM is an 18-item ordinal scale with seven levels ranging from 1 (total dependence) to 7 (total independence), and the best score is 126. The FIM range in this study was from 18 to 126 [30].
- The global functional capacity was assessed with the Modified Barthel Index (BI) [31]. It scores from 0 to 100 (best score).
- Power Hand Grip (bilateral) was a measure of strength performed with a hydraulic dynamometer (Jamar Plus+, Performance Health, Chicago, USA) [32] and adjusted for patient body mass index.
- The Motor Evaluation Scale for Upper Extremity in Stroke (MESUPES) assesses the movements of the upper limb. It consists of two sections, one focusing on the arm and the other on the hand. In the arm section, participants are required to move the affected arm in different positions while supine and seated. The hand tasks evaluate the range of motion and hand orientation when manipulating small objects [33]. MESUPES scores range from 0 to 58, with a higher score indicating better performance.
- Arm disability was assessed with the Quick version of the Disabilities of the Arm, Shoulder, and Hand (Quick-DASH) questionnaire [34]. The Quick-DASH is an 11-item ordinal scale that rates items on a 5-level scale from 1 (no difficulty) to 5 (unable to do). It provides a summative score on a 100-point scale, with 100 indicating the most disability.

### 2.3. Rehabilitation Programme

Eligible patients were randomised to treatment (TG) or control group (CG) in a 1:1 ratio using a simple randomisation procedure (computerised random numbers) conducted independently of the study investigators.

All patients performed the usual rehabilitation programme [35,36] which was adapted to the patient’s needs. A team of specialists (doctors, speech therapists, and physiotherapists) designed a tailored rehabilitation programme for each patient. Rehabilitation began the day after admission. The programme was performed from Monday to Saturday for about six weeks. All patients underwent, on average, 330 min/week of motor rehabilitation (6 days/week), 150 min/week of upper limb intervention (5 days/week), and 200 min/week of speech rehabilitation (4 days/week).

#### Upper Limb Intervention

For specific upper limb rehabilitation, all patients performed the specific intervention consisting of 30 sessions lasting 30 min/day, five days/week, under the supervision of an occupational therapist. Therapy consisted of goal-oriented and highly repetitive training, where all patients performed the following movements:

- For the wrist: radial and ulnar deviation movements, flexion and extension movements, and pronation and supination movements;
- For the hand: movement of opening and closing fingers;
- For the arm: up and down movements, left and right movements, and back and forth movements.

In both groups, the physiotherapist intervened in the patient’s education by teaching them the activity to be performed. While the patients performed the exercises independently, the physiotherapist was always present throughout the activity and only provided assistance upon request.

In the CG, the patient carried out the self-mobilisation without the use of any equipment, helping himself with the healthy limb to carry out the movement when necessary.

**Figure 1:**
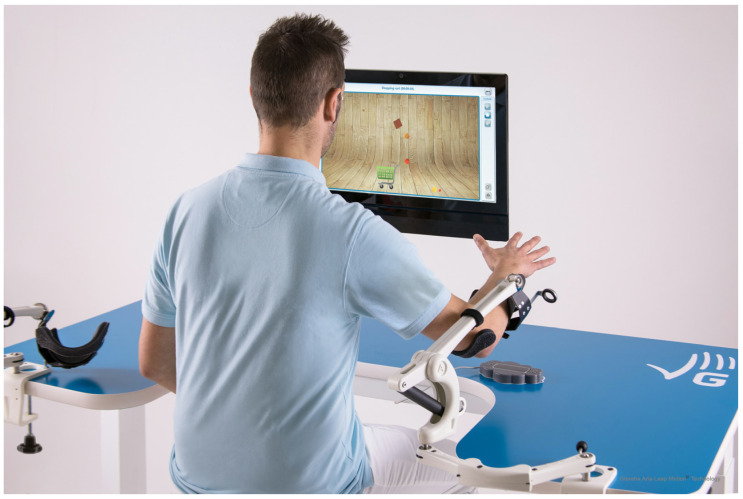
Gloreha Aria (R-Lead).

In the TG, movements were performed using the Gloreha Aria (R-Lead) (Idrogenet, Lumezzane, Italy) [37], a sensor-assisted passive device designed for the motor recovery of impaired upper limbs (Figure 1).

Gloreha Aria (R-Lead) (Idrogenet, Lumezzane, Italy) is equipped with an optical sensor that can measure the patient’s active finger, wrist, and arm movements in space. The active movements are then processed by the software, which uses them as input to perform specific exercises to help patients move their upper limbs and play motivating and challenging motor and cognitive exergames. The level of difficulty of the exercises is automatically adjusted according to the patient’s performance, so that the therapy is always challenging but never frustrating.

The unit is also equipped with two Dynamic Arm Supports, which partially or fully compensate for the weight of the patient’s arms. The ergonomic shape of the table and the automatic height adjustment of the work surface optimise the patient’s position. An integrated touch-screen computer provides a user-friendly control panel for the therapist and an intuitive feedback interface for the patient.

### 2.4. Measures

The feasibility of device use was assessed in terms of the number of patients who completed the programme, side effects (the physiotherapist was required to report any adverse events that occurred during the study in relation to the use of the Gloreha Aria (R-Lead), the time spent by the physiotherapist assisting the patient in the educational phase of activity, and the level of difficulty for the physiotherapist in using the device. This was assessed using a visual analogue scale (VAS) (0 extremely easy–10 extremely difficult). and comparing the mean of the values reported in the first 3 days with the mean of the values reported in the last 27 days.

Concerning the device performance, in all patients from the two groups, we assessed upper limb function with scales at baseline and after 30 sessions of hand rehabilitation performed during hospitalization. The primary evaluation was the Fugl–Meyer Assessment for Upper Extremity (FMA-UE) score, assessed both as an overall motor score and individual items. Secondary evaluations included changes in the following: (1) Global function (Modified BI); (2) FIM; (3) MESUPES; (4) Power Hand Grip; and (5) Quick-DASH.

### 2.5. Statistical Analysis

Statistical analysis was carried out with GraphPad Prism 4, version 4.03. The normality of variables was assessed using the Shapiro–Wilk test. The sample size (*n* = 20) for this pilot study was determined to assess device performance considering a post- and pre-variation of the FMA-UE score of 10 ± 10 in both groups, with α = 0.05 and β = 0.80. For comparisons within-group, all continuous variables were evaluated by the Student’s paired *t*-test, while the Student’s unpaired *t*-test was used for comparisons between-group. Delta changes in all variables T1-T0 (post and pre) for both groups were reported and shown as mean, standard deviation, and confidence intervals at 95%. All *p* values were two-sided and considered significant for values < 0.05.

## 3. Results

Between November 2018 and October 2021, we enrolled 21 patients in our units during hospitalization. Table 1 shows the clinical characteristics of the study groups at admission. No significant differences in demographics or baseline evaluations were observed among the two groups. The hospital length of stay was 60 ± 9 days in the CG and 65 ± 31 days in the TG (*p* = 0.5732).

All the enrolled patients, 10 in the TG and 11 in the CG, completed all hand rehabilitation sessions during their hospital stay without experiencing any adverse events. In TG, the degree of difficulty with managing the device was evaluated daily by the physiotherapist by VAS. The mean VAS score in TG for the first three days was 3.4 ± 1.6 vs. 1.2 ± 1.2 (*p* = 0.0027) for the last 27 days. In the CG group, the mean VAS score for the first three days was 3.1 ± 3.3 vs. 2.1 ± 3.0 (*p* = 0.4657) for the last 27 days. The time commitment of the physiotherapist decreased from 17 ± 11 min in the first 3 days to 6 ± 9 min (*p* = 0.0249) in the last 27 days in TG, and from 28 ± 5 in the first 3 days to 22 ± 12 (*p* = 0.1415). No patient refused the device.

All patients were right-handed, so the treated hand was not always dominant.

**Figure 2:**
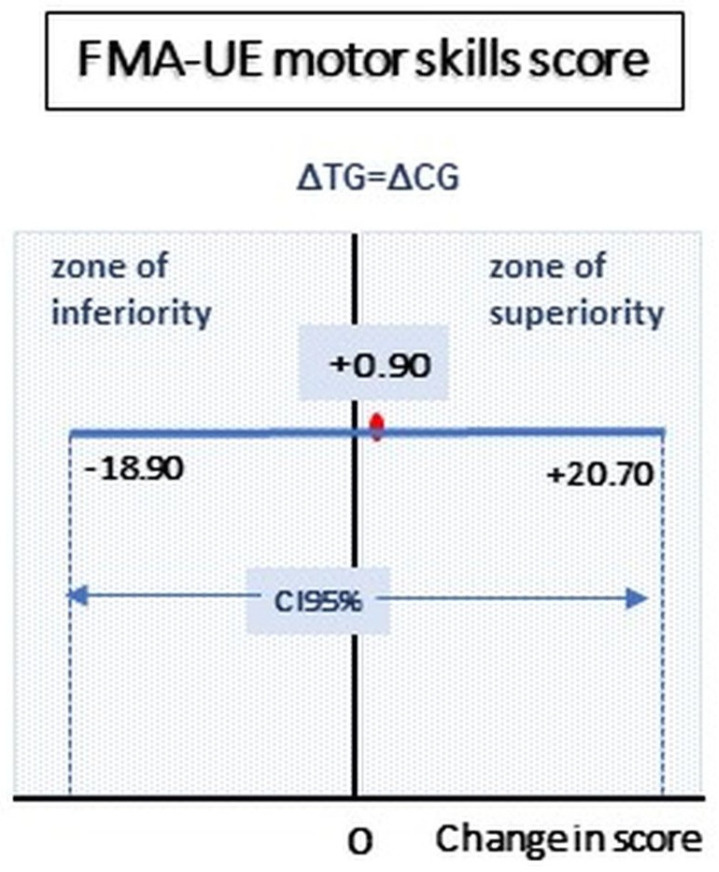
FMA-UE motor skill score, evaluated as the difference between delta changes in TG and CG scores, gives an indication of device performance. Legend: CG: control group, TG: treated group; the red dot on the blue line represents (ΔTG − ΔCG) mean value, CI: confidence interval.

Upper limb motor function improvement was demonstrated by an increase in the FMA-UE score in both groups: from 30.7 ± 18.3 to 42.5 ± 18.4 (*p* = 0.0017) in the CG and from 31.0 ± 13.4 to 43.7 ± 11.4 (*p* = 0.0011) in the TG, respectively (Table 2). The delta changes for FMA-UE (evaluated as ΔTG − ΔCG score, Table 2) and its CI are shown in Figure 2.

All individual motor items (A: upper extremity including shoulder, elbow, and forearm; B: wrist; C: hand grip; and D: coordination/speed) contributed to the improvement in the motor skills FMA-UE over time, with no difference between the two groups (Table 2). Moreover, the joint pain (J) item ameliorated in TG from 20.1 ± 3.8 to 21.8 ± 2.9 (*p* = 0.0220), with scores close to the best value confirming the feasibility of the device use.

All patients in both groups demonstrated significant improvements after intervention also in the secondary outcomes. In particular, as reported in Table 3, the global function capacity (BI) and degree of independence and need of assistance in the basic activities of daily living (FIM) increases over time (both: *p* < 0.001). The Ashworth spasticity index in the two studied areas did not change significantly compared to the baseline in either group after inpatient rehabilitation. The index was respectively 0.47 ± 0.55 in the CG (*p* = 1) and 0.42 ± 0.55 in the TG (*p* = 0.05462) of the wrist, 0.64 ± 0.67 in the CG (*p* = 0.3960) and 0.40 ± 0.52 in TG (*p* = 0.8144) for the below.

In Table 4, we describe the quality of movement performance of the hemiparetic arm and hand in stroke patients measured through the Motor Evaluation Scale for Upper Extremity (MESUPES), the presence and degree of symptoms, as well as the perceived ability to perform functional tasks with the upper limb using the Quick version of the Disabilities of the Arm, Shoulder, and Hand (Quick-DASH) questionnaire, and finally, the grip strength of the injured hand. While the performance quality using MESUPES and grip strength significantly improved at T1 in both groups (both: *p* = 0.010), the Quick-DASH scale showed no statistically significant relevant change. No difference in delta change between groups was confirmed for secondary outcomes (Table 4).

## 4. Discussion

This randomised pilot study studied the use of a sensor-based device, Gloreha Aria (R-Lead), for upper limb rehabilitation in post-stroke patients in addition to the occupational therapy performed by therapists. Gloreha Aria (R-Lead) is a passive sensor-based device for upper limb treatment that offers cognitive exercises and interactive games focusing on free arm, wrist, and hand movements. The patient moves his upper limb in space in the absence of gravity. The set-up is immediate, with nothing to wear on the patient. The device reduces the load on the upper limb without pain, facilitating movement even in patients with reduced upper limb functionality. Therefore, this makes the device more usable even in the early phase after the stroke event.

The Gloreha Aria (R-Lead) programme was feasible, and 100% of patients completed all rehabilitation sessions. From the physiotherapist’s point of view, the first three days required a greater investment of time to teach the correct use of the device, although this was never high. In addition, therapy management with Gloreha Aria (R-Lead) was simple and took little time. The level of difficulty reported by the therapist was low and decreased as the sessions progressed. In the control group, the time spent by the therapist remained higher over time, as did the difficulty expressed, although it was not high.

Concerning the device performance, patients in both groups significantly improved the sensorimotor function measured through the Fugl–Meyer Assessment Upper Extremity (FMA-UE) scale. The FMA-UE is a measure of impairment level that quantifies clinical observations of the stages of motor recovery after a stroke. It is considered the gold standard for assessing upper extremity motor recovery. In the current study, we observed that the post- and pre-improvement in FMA-UE motor skills was significant and similar in both groups. The improvement was higher than the minimal clinically important difference of 9 points suggested by Arya et al. [38] in subacute stroke patients, suggesting clinically important changes in FMA-UE motor skills compared to baseline in these patients over time. Again, the current findings are consistent with the study by Huynh et al. [39], who recently reported that the responsiveness of “a 13-point change on the FMA-UE during the acute to subacute phase of stroke recovery (acute to 6 weeks post stroke) reflects a meaningful change in the recovery of arm motor impairment”.

Concerning FMA-UE single items, the improvement over time was present in almost all the items. However, due to the limited sample, we cannot draw conclusions on the differences between changes in the two groups.

Regarding the secondary evaluations on device performance, the use of the Gloreha Aria (R-Lead) gave a similar performance to hand physiotherapy over time in terms of the quality of movement performance of the hemiparetic arm and hand, as measured by the Motor Evaluation Scale for Upper Extremity (MESUPES), and grip strength of the injured hand, arm, and shoulder. No data from the literature are available for comparison. Instead, the FIM and Barthel Index were not modified; in particular, according to data from Bertani et al. [40], the results of the meta-analysis for FIM showed a high heterogeneity across studies in subacute stroke, in line with the findings of the present pilot study.

The association of upper limb movements with VR support has been shown to be adequate for neurological patients in improving motor function in chronic stroke [41]. Moreover, research has shown that VR systems, particularly those using game-based rehabilitation, can be effective in improving upper limb function and hand mobility in stroke patients [42]. Gamification can motivate patients to actively participate in rehabilitation with enjoyment, which could lead to a better recovery [43]. Gloreha Aria (R-Lead) can provide patients with clear and meaningful feedback coupled with specific goals for occupational therapy sessions. The system’s ability to dynamically adjust the level of difficulty of the exergames based on each patient’s abilities is critical to ensuring that the game remains engaging and conducive to meaningful progress, particularly for patients recovering from stroke who may face numerous challenges and frustrations due to motor impairments [44,45].

The results of this study encourage rehabilitation professionals to incorporate sensor-based technologies such as Gloreha Aria (R-Lead) into their treatment approaches for stroke patients. From an organisational point of view, even more than one unit of such VR devices can be placed in a gym room and managed by one therapist to maximise efficiency and limit the cost of treatments. From a clinical perspective, gamification can increase the effectiveness of the session by stimulating neuroplasticity and promoting patient engagement and motivation, which cannot be taken for granted given the typically long recovery period after stroke [43,44,45].

However, stimulation of neuroplasticity remains to be specifically proven. Actually, the Gloreha Aria (R-lead) has a section dedicated to assessing movements of finger flexion and extension, wrist flexion and extension, wrist pronation and supination, ulnar radial deviation of the wrist, and whole arm movements (up and down, left and right, forward and backward). The device does not provide quantitative data but only a simple qualitative assessment that allows the clinician to have some information about the patient’s progress. In this regard, this study does not provide information on the psychological/neuropsychological aspects of device performance, which remains an issue to be investigated.

In terms of future developments, it would be interesting on one hand to implement a system that, through the use of the optical sensor of the Gloreha Aria (R-lead) device, perhaps even with the integration of other sensors (for example, EMG sensors), would be able to provide an assessment of the upper limb, as proposed by Tahir et al. [46], and on the other hand, it would be interesting to consider the study of neuropsychological aspects [47,48], which could represent a vast scenario from which patients could benefit.

There are some study limitations to consider. This pilot study was conducted on a limited number of patients post- and pre-comparison and in a single centre only. Indications from delta changes (ΔTG − ΔCG) should be treated with caution and need to be confirmed in a further study of efficacy in a larger population. Furthermore, this device does not apply to patients with an index of hand Ashworth spasticity ≥ 3, limiting its application field.

## 5. Conclusions

The present study confirms the feasibility of Gloreha Aria (R-Lead) employment and gives promising indications for UL motor rehabilitation as measured by FMA-UE. The device’s use of virtual reality assists physiotherapists, allowing them to devote more time to patients who require face-to-face intervention or cannot be treated by the robot per se. However, the effectiveness of the device’s performance remains to be demonstrated. Further studies are needed to explore the extent of implementation and effectiveness of AR combined with conventional rehabilitation in a larger population.

## Figures and Tables

**Table 1:** Clinical characteristics of the study group at admission.

	Control Group (*n* = 11)	Treatment Group (*n* = 10)	*p*-Value
Males, *n* (%)	9 (82%)	8 (80%)	
Age, years	71 ± 13	68 ± 15	0.8639
BMI, kg/m^2^	24.2 ± 3.9	26.8 ± 3.1	0.1464
Time from acute event to inclusion, days	12.7 ± 4.9	16.6 ± 5.9	0.0611
Ischemic stroke, *n* (%)	8 (73%)	5 (50%)	0.5344
Haemorrhagic stroke, *n* (%)	3 (27%)	5 (50%)	
Paretic side:			
- Right, *n* (%)	6 (54%)	5 (50%)	0.8188
- Left, *n* (%)	5 (46%)	5 (50%)	
Ashworth spasticity index of the following:			
- Wrist, score	0.47 ± 0.55	0.54 ± 0.74	0.8578
- Elbow, score	0.47 ± 0.71	0.44 ± 0.57	0.9713
FIM, score	66.9 ± 10.2	65.7 ± 24.5	0.8053
Barthel Index, score	31.1 ± 12.7	32.5 ± 21.1	0.6723
Motor Skill FMA-UE, score	30.7 ± 18.3	31.0 ± 13.4	0.8602
Quick-DASH, score	46.1 ± 25.7	55.9 ± 16.5	0.5727
MESUPES, score	22.4 ± 13.0	18.4 ± 12.9	0.6219
Grip injured hand, score	10.0 ± 8.3	8.6 ± 4.0	0.8603
Grip healthy hand, score	24.9 ± 11.0	30.2 ± 11.6	0.2599

Legend: Data were expressed as number (%) or mean ± SD. Abbreviations: BMI, Body Max Index; FIM, Functional Independence Measure; Barthel Index; Modified Barthel Index; FMA-UE, Fugl–Meyer Assessment Upper Extremities; Quick-DASH, Quick version of the Disabilities of the Arm, Shoulder, and Hand questionnaire; MESUPES, Motor Evaluation Scale for Upper Extremity in Stroke.

**Table 2:** Comparison of the Fugl–Meyer Assessment Upper Extremity (FMA-UE) to measure sensorimotor function post- (T1) and pre- (T0) intervention.

FMA Single Items	Control Group (Mean ± SD)			Treatment Group (Mean ± SD)			Changes between Post- and Pre-Intervention [Mean ± SD (95% CI)]		
	Pre	Post	*p* Value	Pre	Post	*p* Value	ΔCG	ΔTG	*p* Value
A. Upper Extremity	17.6 ± 9.5	23.4 ± 10.1	0.0018	19.1 ± 6.4	25.1 ± 5.5	0.0007	5.8 ± 4.6 (2.7~8.9)	6.0 ± 3.7 (3.3~8.7)	0.9644
B. Wrist	3.5 ± 3.9	5.5 ± 2.8	0.0645	3.9 ± 3.01	5.9 ± 2.8	0.0059	1.9 ± 3.1 (−0.1~3.9)	2.0 ± 1.8 (0.7~3.3)	0.8602
C. Hand	6.6 ± 5.4	9.7 ± 4.8	0.0045	5.9 ± 4.7	10.1 ± 3.5	0.0089	3.1 ± 2.8 (1.2~4.9)	4.2 ± 4.0 (1.3~7.1)	0.4724
D. Coordination /Speed	2.9 ± 2.2	3.9 ± 1.9	0.0127	2.1 ± 1.7	2.6 ± 2.2	0.3221	1 ± 1.1 (0.3~1.7)	0.5 ± 1.5 (−0.6~1.6)	0.4790
(A–D) FMA motor skills, Figure 2	30.7 ± 18.3	42.5 ± 18.4	0.0017	31.0 ± 13.4	43.7 ± 11.4	0.0011	11.8 ± 9.2 (5.6~18.0)	12.7 ± 8.6 (6.6~18.8)	0.8664
H. Sensation	10.6 ±2.4	11.6 ± 0.9	0.1688	8.2 ± 4.5	10 ± 2.5	0.1309	1 ± 2.2 (−0.5~2.5)	1.8 ± 3.4 (−0.7~4.3)	0.6745
J. Passive Joint motion	16.2 ± 6.6	18.8 ± 6.8	0.0193	20.2 ± 3.7	21.3 ± 2.9	0.0399	2.6 ± 3.1 (0.5~4.7)	1.1 ± 1.5 (0.1~2.1)	0.1114
J. Joint Pain	23.4 ± 1.8	23.2 ± 2.7	0.8639	20.1 ± 3.8	21.8 ± 2.9	0.0220	−0.2 ± 3.4 (−2.5~2.1)	1.7 ± 2.0 (0.3~3.1)	0.1551

Legend: Data were reported as mean ± SD, or mean ± SD, with a confidence interval (CI 95%). Abbreviations: FMA-UE, Fugl–Meyer Assessment Upper Extremity [Single items: A. Upper Extremity (best value 36); B. Wrist (best value 10); C. Hand (best value 14); D. Coordination/Speed (best value 6); A–D. UE_motor skill (best value 66); H. Sensation (best value 12); J. Passive Joint motion (best value 24); J. Joint Pain (best value 24)].

**Table 3:** Comparison of the assessments of the global functional capacity and the functional independence measure post- and pre-intervention in the two groups.

		Barthel Index	FIM
Control Group	Pre	31.1 ± 12.7	66.9 ± 10.2
	Post	71.8 ± 20.1	103.5 ± 15.6
Within group *p* value		0.0002	<0.0001
Treatment Group	Pre	32.5 ± 21.1	65.7 ± 24.5
	Post	67.4 ± 21.1	98.0 ± 23.6
Within group *p*-value		0.0005	<0.0001
Changes between groups	ΔCG (T1 − T0)	40.7 ± 24.0 (24.6~56.8)	36.6 ± 15.4 (26.3~47.0)
	ΔTG (T1 − T0)	34.9 ± 21.1 (19.8~50.0)	32.3 ± 12.0 (23.8~40.8)
Between groups *p*-value		0.5073	0.0907

Legend: Data were reported as mean ± SD. Abbreviations: FIM, Functional Independence Measure; Barthel Index, Modified Barthel Index; CI, confidence interval.

**Table 4:** Comparison of the assessments of the paretic side post- and pre-intervention in the two groups.

		Quick-DASH	MESUPES	Power Hand Grip
Control Group	Pre	46.1 ± 25.7	22.4 ± 13.0	10.0 ± 8.3
	Post	43.2 ± 28.9	31.7 ± 10.6	15.8 ± 10.5
Within group *p* value		0.7919	0.010	0.010
Treatment Group	Pre	55.9 ± 16.5	18.4 ± 12.9	8.6 ± 4.0
	Post	45.2 ± 18.2	27.5 ± 13.9	17.2 ± 8.6
Within group *p*-value		0.1377	0.010	0.0201
Changes between groups	ΔCG (T1-T0)	−2.9 ± 35.7 (−26.9~21.1)	9.4 ± 8.7 (3.5~15.2)	5.7 ± 5.3 (2.1~9.3)
	ΔTG (T1-T0)	−10.7 ± 20.7 (−25.5~4.1)	9.1 ± 8.8 (2.8~15.4)	8.7 ± 9.7 (1.7~15.6)
Between groups *p*-value		0.6504	0.8603	0.5068

Legend: Data were reported as mean ± SD and confidence interval (CI 95%). Abbreviations: MESUPES, Motor Evaluation Scale for Upper Extremity in Stroke; Quick-DASH, Quick version of the Disabilities of the Arm, Shoulder, and Hand questionnaire.

## Data Availability

Data are available upon request to the corresponding author.
